# A source of resistance against yellow mosaic disease in soybeans correlates with a novel mutation in a resistance gene

**DOI:** 10.3389/fpls.2023.1230559

**Published:** 2023-11-24

**Authors:** Saleem Ur Rahman, Ghulam Raza, Rubab Zahra Naqvi, Evan McCoy, Muhammed Hammad, Peter LaFayette, Wayne Allen Parrott, Imran Amin, Zahid Mukhtar, Abdel-Rhman Z. Gaafar, Mohamed S. Hodhod, Shahid Mansoor

**Affiliations:** ^1^ National Institute for Biotechnology & Genetic Engineering College, Pakistan Institute of Engineering and Applied Sciences (NIBGE-C, PIEAS), Faisalabad, Pakistan; ^2^ Department of Allied Health Sciences, Pak-Austria Fachhochscule-Institute of Applied Sciences and Technology (PAF-IAST), Mang, Haripur, Khyber Pakhtunkhwa, Pakistan; ^3^ Institute of Plant Breeding, Genetics & Genomics, University of Georgia, Athens, GA, United States; ^4^ Department of Botany and Microbiology, College of Science, King Saud University, Riyadh, Saudi Arabia; ^5^ Department of Biotechnology, October University for Modern Sciences and Arts, 6th of October City, Egypt; ^6^ Jamil ur Rehman Center for Genome Research, International Center for Chemical and Biological Sciences (ICCBS), University of Karachi, Karachi, Pakistan

**Keywords:** yellow mosaic disease, soybean, screening, resistance source, NBG-soybean

## Abstract

Yellow mosaic disease (YMD) is one of the major devastating constraints to soybean production in Pakistan. In the present study, we report the identification of resistant soybean germplasm and a novel mutation linked with disease susceptibility. Diverse soybean germplasm were screened to identify YMD-resistant lines under natural field conditions during 2016-2020. The severity of YMD was recorded based on symptoms and was grouped according to the disease rating scale, which ranges from 0 to 5, and named as highly resistant (HR), moderately resistant (MR), resistant (R), susceptible (S), moderately susceptible (MS), and highly susceptible (HS), respectively. A HR plant named “NBG-SG Soybean” was identified, which showed stable resistance for 5 years (2016-2020) at the experimental field of the National Institute for Biotechnology and Genetic Engineering (NIBGE), Faisalabad, Pakistan, a location that is a hot spot area for virus infection. HS soybean germplasm were also identified as NBG-47 (PI628963), NBG-117 (PI548655), SPS-C1 (PI553045), SPS-C9 (PI639187), and cv. NARC-2021. The YMD adversely affected the yield and a significant difference was found in the potential yield of NBG-SG-soybean (3.46 ± 0.13^a^ t/ha) with HS soybean germplasm NARC-2021 (0.44 ± 0.01^c^ t/ha) and NBG-117 (1.12 ± 0.01^d^ t/ha), respectively. The YMD incidence was also measured each year (2016-2020) and data showed a significant difference in the percent disease incidence in the year 2016 and 2018 and a decrease after 2019 when resistant lines were planted. The resistance in NBG-SG soybean was further confirmed by testing for an already known mutation (SNP at 149^th^ position) for YMD in the *Glyma.18G025100* gene of soybean. The susceptible soybean germplasm in the field was found positive for the said mutation. Moreover, an ortholog of the *CYR-1* viral resistance gene from black gram was identified in soybean as *Glyma.13G194500*, which has a novel deletion (28bp/90bp) in the 5`UTR of susceptible germplasm. The characterized soybean lines from this study will assist in starting soybean breeding programs for YMD resistance. This is the first study regarding screening and molecular analysis of soybean germplasm for YMD resistance.

## 1 Introduction

Yellow mosaic disease (YMD) is one of the major devastating diseases that severely hampers soybean production. The disease is mainly caused by the mungbean yellow mosaic India virus (MYMIV), mungbean yellow mosaic virus (MYMV), horsegram yellow mosaic virus (HgYMV), and dolichos yellow mosaic virus **(**DoYMV). These viruses cause characteristic symptoms of yellow mosaic patterns on the leaf surfaces and are collectively named legume yellow mosaic viruses (LYMVs) (Qazi et al., 2007; Ilyas et al., 2009; Rahman et al., 2023a). Among LYMVs, HgYMV and DoYMV are rarely found, while MYMV and MYMIV are very common and infect many important legume crops (Maruthi et al., 2006; Rahman et al., 2023a). Previously, both MYMIV and MYMV species were found in India, while in Pakistan, MYMIV was the most frequent species found to infect major legume crops (Ilyas et al., 2009; Ilyas et al., 2010). However, recently a comprehensive study was conducted and found that MYMIV and MYMV have an equal role in causing infection in soybean cultivars in Pakistan (Rahman et al., 2023a; Rahman et al., 2023b). The exact data on yield loss due to YMD is not available, as the incidence of YMD varies in different locations and also varies for different crops (Varma and Malathi, 2003). In 1996, YMD caused a significant yield loss in soybean production, of approximately 105,000 metric tonnes. It has been reported that if the disease appears in the early stage of plant growth, the yield loss reaches up to 100% (Wrather et al., 1997; Kitsanachandee et al., 2013). Official reports on YMD from soybeans are lacking in Pakistan as soybean was only recently grown as a major legume in the country, however, anecdotal evidence from virologists suggests the disease is very common.

Identification of resistant soybean germplasm is a method of choice to prevent soybean cultivars from contracting YMD. In India, many resistant and susceptible soybean cultivars have been identified for YMD (Ram et al., 1984; Lal et al., 2005; Rani et al., 2017), but in Pakistan, no resistant cultivars have been identified or tested. Identification of virus-resistant germplasm is also the first step toward the identification of resistant (R) genes. These R genes have an important role in disease control. In 2012, the marker-assisted breeding technique was used for the identification of R genes and many genes have been identified that were found linked with YMD, such as the *CYR-1* gene in black gram (Maiti et al., 2012). The *CYR-1* gene was also found completely linked with MYMIV resistance in urdbean and mungbean (Maiti et al., 2011). In 2016, it was reported that the recessive form (*cyr-1*) of the *CYR-1* gene is a susceptibility factor for YMD in black gram (Patel et al., 2016). In urdbean, RGA-1, CEDG180, ISSR811, and YMV-1 were found to be closely linked with MYMIV resistance (Basak et al., 2005; Souframanien and Gopalakrishna, 2006; Gupta et al., 2013). In mungbean and black gram, the resistance gene analog (RGA) marker has been found to be linked with MYMV resistance. However, very little information is available on YMD-resistance genes in soybeans, except single nucleotide polymorphism (SNP) in an LRR-like protein kinase gene (chromosome 18; Glyma18g02850), which was found to be associated with soybean susceptibility to MYMIV (Yadav et al., 2015).

Studies regarding natural resistance sources for YMD and resistance genes from soybeans in Pakistan are lacking and the subject is in dire need of investigation. This study highlights the identification of both resistant and susceptible soybean germplasm and markers for YMD resistance in soybean cultivars, which will be used for the screening of resistant sources in soybean breeding programs in the future.

## 2 Materials and methods

### 2.1 Screening of soybean germplasm for YMD

A total of 1,007 soybean accessions were screened from 2016 to 2020. These accessions were acquired from the Plant Genetic Resources Institute (PGRI, Pakistan) and the Agriculture Research Service of the United States Department of Agriculture (USDA-ARS), USA. Local cultivars were provided by the National Agriculture Research Center (NARC), Islamabad, and Agriculture Research Center (ARS), Swat, Khyber Pakhtunkhwa, Pakistan as well as Ayub Agriculture Research Institute (AARI), Faisalabad, Pakistan. The imported germplasm, along with locally adapted cultivars, (Faisal soybean, NARC-2021, cv. Ajmeri) were grown in three replicates in single row plots that were 4.5 m long and were located at an experimental field, NIBGE, Faisalabad (31°25'0"N 73°5'28" E). The trial location is a regional hub for begomoviruses of crops commonly grown in the area, such as cotton and mungbean (Habib et al., 2007; Ilyas et al., 2010). The plant-to-plant distance was 10 cm and the row-to-row distance was 30 cm. The experiment was performed during the autumn seasons (from August to November) of the years 2016-2020. Recommended agronomic practices were performed for soybean management. The field was plowed two to three times before seed sowing. The recommended seed rate of 85 kg/ha of soybean was used. To achieve high nodulation and better nitrogen fixation, seeds were inoculated with plant growth-promoting rhizobacteria (PGPR) in the form of *Bradyrhizobium japonicum* (10 g per kg of soybean seeds). Recommended fertilizer rates of 25:60:50 kg/ha of Nitrogen (N), Phosphorus (P), and Potassium (K) were applied, respectively, by utilizing commercially available fertilizers (Urea, DAP, and SOP). During seedbed preparation, a full dose of P and K and a half dose of N were applied as basal doses. The remaining N was used at the flowering stage. To keep the crop free from weeds, the pre-emergence herbicide, Dual Gold (S-Metolachlor; Syngenta, Switzerland), at a concentration of 960g/L was used. Before sowing, the seeds were also treated with an antifungal, Hombre Ultra (Imidacloprid; Bayer, Germany). All the agronomic practices were kept uniform for all treatments except the soybean cultivars under study. The soybean crop was irrigated five to six times during the season. The field was routinely visited after sowing to document the appearance of symptoms of YMD. When YMD emerged, such as yellow mosaic spots on the leaf surface, the infection percentage was measured. The disease severity index (DSI)/percent infection (PI) of each cultivar was recorded. For DS, six different groups, namely, highly resistant (HR), resistant (R), moderately resistant (MR), moderately susceptible (MS), susceptible (S), and highly susceptible (HS), were formed as previously described (Islam et al., 2010) as shown in **Table 1**. HR represents high resistance to viruses having no symptoms. The percentage scale shows the plant infection severity of leaves and plant area affected. The DSI was calculated using the following formula (Habib et al., 2007):

**Table 1:** Criteria for percent infection of YMD in soybean germplasm.

Percent Infection	Disease Severity/Scale	Infection Category	Reaction Group
No symptoms	0	Highly resistant	**HR**
1-10%	1	Resistant	**R**
11-20%	2	Moderately resistant	**MR**
21-30%	3	Moderately susceptible	**MS**
31-50%	4	Susceptible	**S**
More than 50%	5	Highly susceptible	**HS**


$$individual\;DSI=\frac{\text{Sum of individual plant rating}}{\text{Total number of observed plants}}]\times 100$$

**Figure 1:**
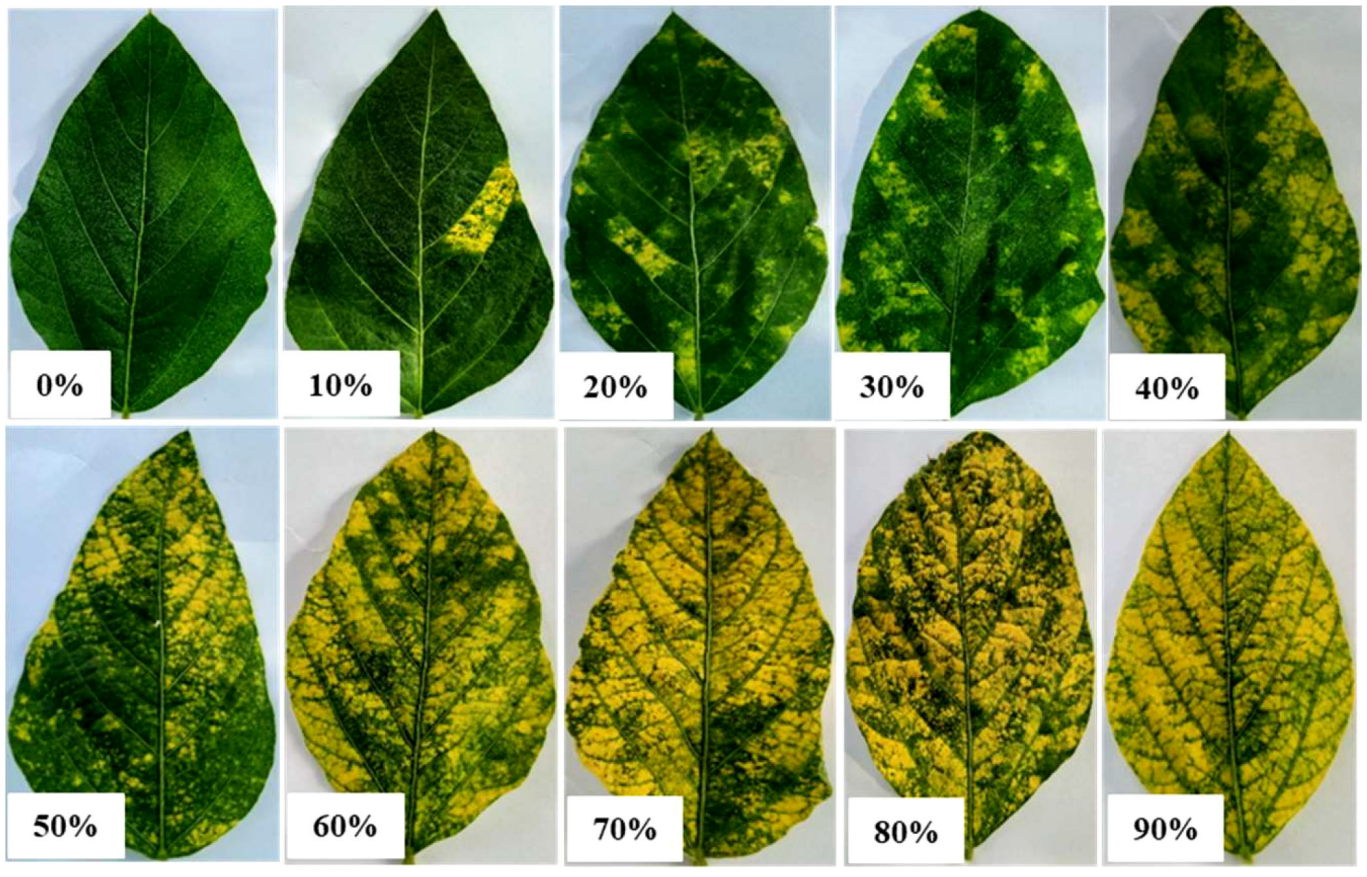
Representation of YMD severity percentage on soybean leaf.


Where DSI is the disease severity index, the disease rating is 0-5 as reported (Habib et al., 2007), and the individual plant rating was based on the symptoms of the leaves of each plant affected by the disease (**Figure 1**). The overall mean of disease rating for individual cultivars was recorded for the entire period (2016-2020). The percentage of disease incidence (PDI) was also measured by selecting the diseased (YMD) plants. In this case, the plants were classified as diseased or healthy irrespective of symptom severity. Several plants were randomly recorded at five positions in the entire field and classified as YMD or healthy. For each position, data from multiple plants were recorded. A total of 100 plants were classified in the entire field. The PDI was calculated using the following formula:

$$PI\;=[\frac{\text{Number of YMD plants}}{\text{Total number of observed plans}}]\times 100$$

### 2.2 Molecular characterization of viruses causing YMD

For the identification of begomoviruses causing YMD in soybeans, both symptomatic (susceptible) and asymptomatic (resistant) samples were collected, and the DNA was extracted using the modified CTAB method (Doyle and Doyle, 1987). The viral DNA was amplified using the primer pair MYMIV-F/MYMIV-R and MYMV-F/MYMV-R (**Table S-2**). A total of 10 PCR amplified products (MYMIV and MYMV) with respective sizes (~2.6-2.8 kb) were purified from agarose gel and cloned in a pTZ57R/T vector (Thermo Scientific, USA). The confirmed clones were sequenced using the Applied Biosystems 3730XL DNA sequencer. These clones were sequenced with M13 forward and M13 reverse primer pair, and then by primer walking strategy to get the complete sequence of each clone in both directions. Sequences were analyzed using Lasergene (DNAStar Inc.), and reads were assembled in SeqMan (Lasergene, DNAStar Inc., Madison, USA). After trimming the vector portion, a consensus contig was saved and analyzed. The sequences were submitted to the GenBank of NCBI (MN885463 and MK098184).

### 2.3 Confirmation of known SNP in soybean germplasm

For the confirmation of the already known resistant gene, *Glyma.18G025100* in soybean (Yadav et al., 2015), highly resistant and highly susceptible genotypes of soybean in Pakistan were selected. The total genomic DNA was extracted using a modified CTAB method (Doyle and Doyle, 1987) and the DNA was amplified using the primer pair: 18G025100-F: TCGTACTCACGAAGGTGGA; 18G025100-R: AATGCGTTCTGAAGCTGTCC (Yadav et al., 2015). The PCR products with the expected size (~2.6-2.8 kb) were gel-eluted and sequenced using the Applied Biosystems 3730XL DNA sequencer. The sequencing results were cleaned and compared using the BLASTn search tool in the NCBI data bank.

### 2.4 Confirmation of *CYR-1* ortholog in soybean

To identify the ortholog of the black gram *CYR-1* gene (Patel et al., 2016) in soybean, the complete sequence of the gene was retrieved from the Phytozome database (https://phytozome-next.jgi.doe.gov/), and BLASTP search engine was used to find the most similar sequences in the soybean genome. The most identical sequences of the predicted genes in soybeans were picked, which were further confirmed by wet lab experiments. The total DNA was extracted using a modified CTAB method (Doyle and Doyle, 1987) and amplified using a diverse range of overlapping primers (**Table S-3**) to sequence the complete gene. These overlapping primers were based on the predicted gene that followed the chromosome-walking strategy. These primers were designed in the available Geneious bioinformatics software. Overlapping primers were applied on both susceptible and resistant soybean cultivars identified in the present study. The PCR products were resolved using gel electrophoresis, purified and Sanger sequenced.

### 2.5 *In silico* analysis of 5`UTR and CYR-1 protein from resistant and susceptible germplasm

The transcription factor binding sites (TFBSs) were detected in both 5`UTR regions from the *CYR-1* gene from resistant and susceptible germplasm by using PlantPAN3.0 (http://plantpan.itps.ncku.edu.tw/). The ProtParam tool at Expasy (https://web.expasy.org/protparam/) was employed to identify the differences in CYR-1 protein from resistant and susceptible germplasm. The protein sequences of CYR-1 protein from resistant and susceptible germplasm were subjected to protein structure prediction and structure-based alignment by I-TASSER (https://zhanggroup.org/I-TASSER/) and TM-align (https://zhanggroup.org/TM-align/). Protein structures were visualized by PyMOL2.5 (https://pymol.org/2/). The interaction of both the CYR-1 gene from resistant germplasm and the CYR-1 gene from susceptible germplasm with the MYMIV viral coat protein was checked using PSOPIA (https://mizuguchilab.org/PSOPIA/) and ISLAND (https://island.pythonanywhere.com/).

### 2.6 Statistical analysis

The data were analyzed using a one-way analysis of variance (ANOVA) and the Tukey test was applied at α =0.05 (95% interval) using GraphPad Prism 6 (https://graphpad-prism.software.informer.com/6.0/).

## 3 Results

### 3.1 Evaluation of soybean germplasm for YMD

**Figure 2:**
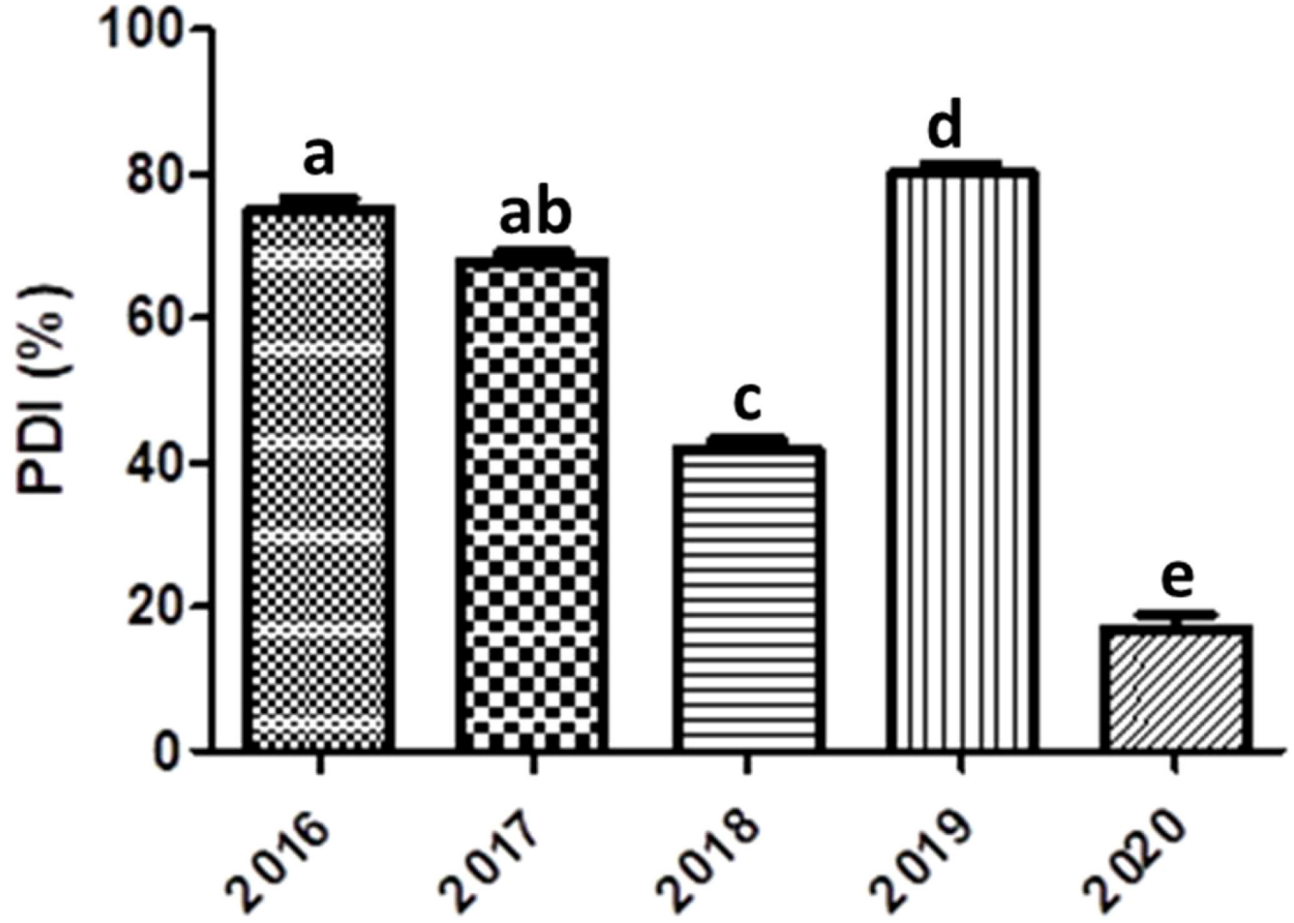
Year-wise YMD incidence (%) in soybeans. Lower case letters show significant difference.

**Figure 3:**
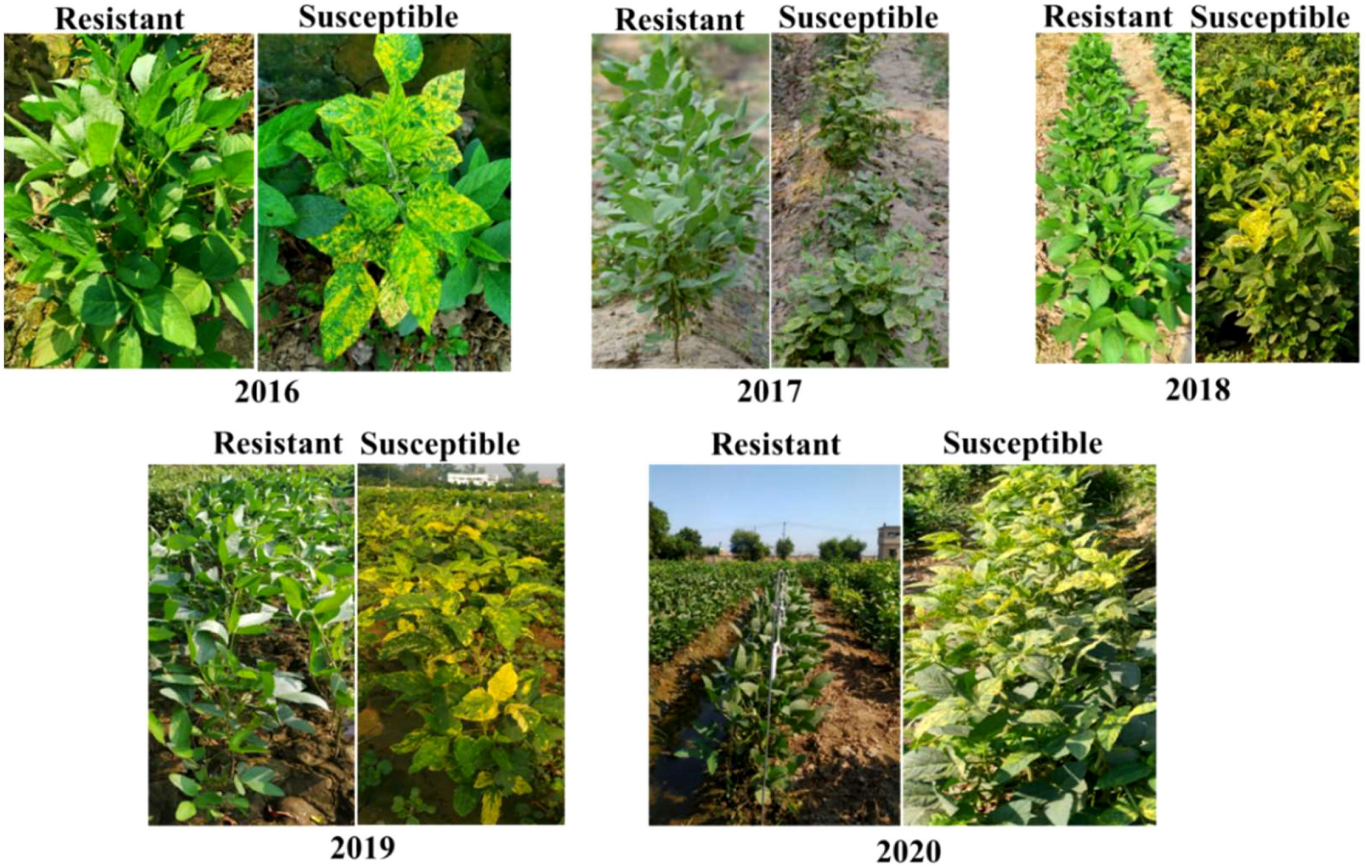
Resistant and susceptible soybean germplasm for YMD tested in 2016-2020.

**Figure 4:**
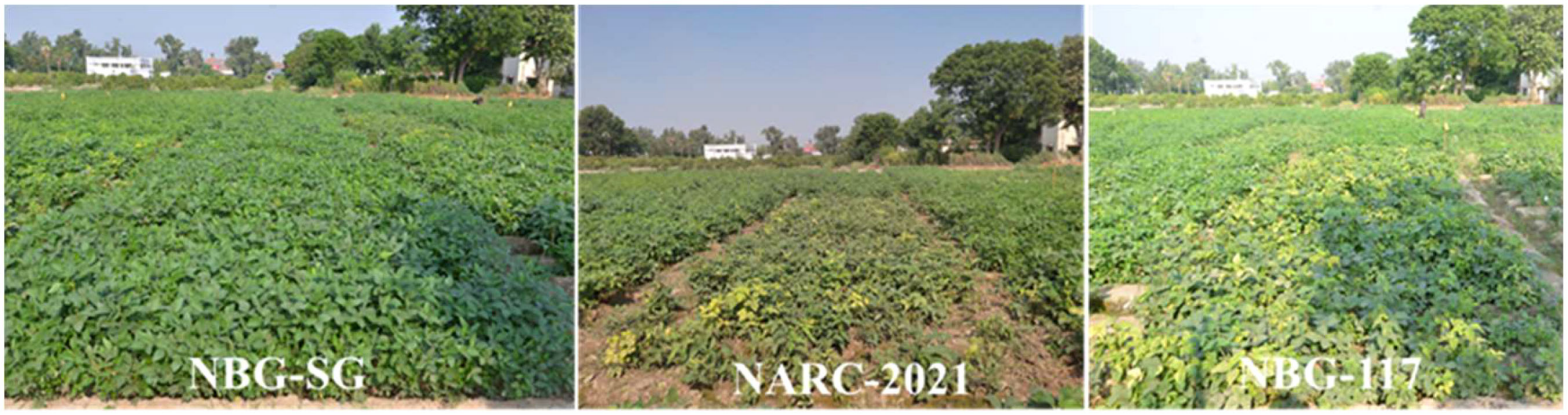
Pictorial view of resistant and susceptible soybean germplasm under natural field conditions.

The soybean accessions (**Table S-1**) were evaluated during five successive years 2016-2020 in the autumn season (from August to November). In the year 2016, out of 128 entries, 18 entries found HR and 30 were HS (**Table 2**). The disease severity index was higher in the year 2016 (**Figure 2**). The HR and HS entries proceeded further along with those accessions having high yield and resistance to YMD. There were more resistant lines in 2019 as crosses and mutants were included, which increased the resistance gene pool further. The individual leaf disease severity scale was converted to individual plants and identified HS and HR soybean germplasm (**Figure 3**). A highly resistant plant, selected from cv. Jack (PI540556) and named SG-soybean, was identified in the year 2016. The resistance in this line was stable in the entire growing period (2016-2020). The SG-soybean line showed complete resistance with no viral symptoms (**Figure 4**), while the approved cultivars, such as cv. Ajmeri and NARC-2021, were found to be HS. The accession NBG-117 (PI548655) and exotic genotypes were also found to be susceptible. The cv. NARC-2021 was previously recorded as NARC-16, which was approved recently in the year 2023 for general cultivation in Khyber Pakhtunkhwa, Pakistan. The incidence of disease in susceptible cultivars was more in check cultivars: cv. Ajmeri and NARC-2021 (**Figure 4**). The persistent nature of these cultivars to virus resistance showed that the susceptibility and resistance in these cultivars were stable (**Table 3**). The SG-soybean line was not only resistant to YMD but was resistant to multiple viruses. To have a complete picture of each cultivar, the reaction of each cultivar in each year and the overall mean reaction are summarized in **Table 3**. The YMD incidence was measured in the years 2016-2020 (**Figure 2**). The disease incidence was found to be very high (**Figure 2**) in 2016 and 2019, while it was significantly low (*P*< 0.05) in the years 2018 and 2020.

**Table 2:** Number of YMD resistant and susceptible soybean genotypes during autumn (2016-2020).

Reaction	Number of Genotypes					
	2016	2017	2018	2019	2020	Total
HR	18	01	01	04	1	25
R	23	00	10	32	6	71
MR	11	00	32	56	11	110
MS	12	01	47	79	16	155
S	34	06	37	127	19	223
HS	30	28	174	160	31	423
**Total**	**128**	**36**	**301**	**458**	**84**	**1007**

**Table 3:** Agronomic performance of highly resistant and susceptible soybeans.

Genotype	DF	DM	PH (cm)	PPP	SPP	GY (t/ha)
**SG-soybean**	41 ± 3.0^a^	98 ± 2.0^a^	37.3 ± 2.0^a^	70.6 ± 3.0^a^	2.86 ± 0.15^a^	3.46 ± 0.13^a^
**NARC-2021**	65 ± 2.6^c^	91.6 ± 3.5^a^	38.3 ± 7.2^a^	93.6 ± 5.5^b^	2.93 ± 0.05^a^	0.44 ± 0.01^c^
**NBG-117**	40 ± 2.5^a^	102.3 ± 2.5^ab^	62.6 ± 8.0^ab^	127.3 ± 12.0^bc^	2.96 ± 0.05^a^	1.12 ± 0.01^d^

The small letters "a-c" etc shows the significant difference.

### 3.2 Yield and yield-linked traits

The yield of the resistant SG-Soybean line was higher (3.46 ± 0.13a t/ha) than that of the susceptible NARC-2021 (0.44 ± 0.01c t/ha) and NBG-117 (1.12 ± 0.01d t/ha) (**Table 3**). The increase in SG soybean was ~7.9 times that of NARC-2021 and ~3 times that of NBG-117 (**Table 3**). Although the SPP and PPP of HS germplasm (NARC-2021 and NBG-117) were high, the germination growth was badly affected by YMD in these cultivars, so the number of plants in these germplasm was lower as compared to the SG-soybean line; hence the yield of these HS soybean germplasm was less than that of the SG-soybean line (**Table 3**).

### 3.3 Molecular analysis for YMD

As the YMD is caused by both MYMIV and MYMV, to confirm the YMD for both the viral strains, the susceptible and resistant cultivars were evaluated (**Table 2**). All the YMD symptomatic plants were positive for MYMIV/MYMV. The sequences of MYMIV and MYMV identified in the field were submitted in NCBI and are available under accession nos. MN885463 and MK098184, respectively.

### 3.4 Confirmation of known SNP in soybean resistant gene

**Figure 5:**
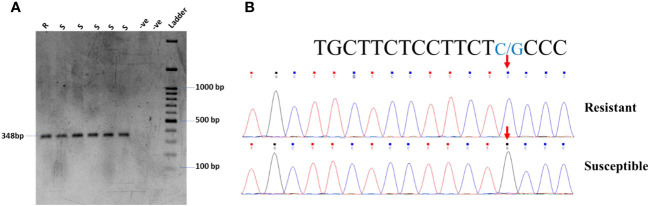
**(A)** PCR amplification of resistant and susceptible germplasm for gene *Glyma.18G25100* validation. **(B)** Chromatogram of *Glyma.18G25100* showing G/C transversion in soybean germplasm.

In the current study, the highly resistant and susceptible germplasm of soybeans in Pakistan were checked for the known SNP in the *Glyma.18G025100* gene on chromosome 18. The primer pair: 18G025100-F: TCGTACTCACGAAGGTGGA and 18G025100-R: AATGCGTTCTGAAGCTGTCC was used for the amplification of the gene carrying the SNP for C to G transversion. The gel electrophoresis confirmed the expected fragment size (**Figure 5A**). However, the PCR product was further confirmed by Sanger sequencing, which found the same C to G transversion, as shown in **Figure 5B**.

### 3.5 Confirmation of the *CYR-1* gene ortholog in soybean

**Figure 6:**
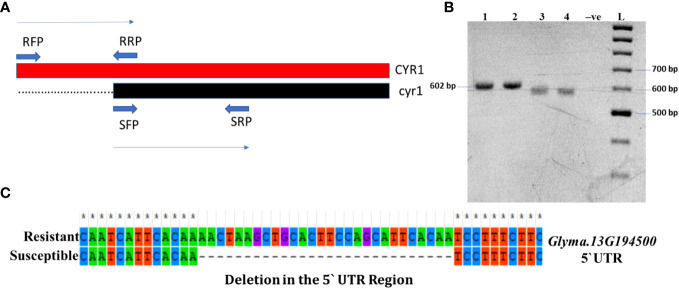
**(A)** Representation of overlapping primers designed to amplify complete *CYR-1* gene; *Glyma.13G194500* **(B)** PCR amplification of *CYR-1* gene; *Glyma.13G194500* **(C)** Alignment of *Glyma.13G194500* of YMD resistant and susceptible soybean. The symbol * shows homology.

To identify the ortholog of the *CYR-1* gene in soybean, the sequence of the *CYR-1* gene was aligned with the soybean genome. The sequence of the *CYR-1* gene of black gram showed 65% identity with the soybean gene *Glyma.13G194500*. The overlapping primers were designed to cover the whole gene with 100 bp apart from the gene covering 5` UTR and 3` UTR (**Table S-3**; **Figure 6A**). The 128 amino acid deletion was not identified in the closely related gene, however, in one susceptible variety (Minsoy), a deletion (28bp) in the 5` UTR was identified. This deletion was also observed when resolved on agarose gel (**Figure 6B**). The deletion was confirmed by Sanger sequencing, which was identified in 5` UTR of *Glyma.13G194500* (**Figure 6C**).

## 4 Discussion

Soybean is one of the most important crops grown in many countries (Rathore et al., 2021). However, Pakistan grows soybeans in a negligible area. Many reasons lead to the low cultivation and production of this crop in the country. Among these, YMD is the main problem in soybean production in the region, based on typical symptoms of YMD observed in the field in Pakistan. YMD also has an important role and impact on the production of mungbean in India as well as in Pakistan (Ilyas et al., 2009; Nair et al., 2017; Ashok, 2018). In India, this disease not only infects mungbean, but also poses a serious threat to other legume crops as well as to soybean production (Gazala et al., 2013). However, India has applied multiple approaches to find ways to control YMD in soybean crops, such as producing resistant cultivars (Mishra et al., 2020). However, despite the dire need for the cultivation of soybeans in Pakistan, there are no reports available regarding cultivars of soybeans resistant to YMD in the country.

Multiple approaches are applied for the control of begomoviruses that cause multiple diseases in plants. One such method is to control their vectors, such as whiteflies, which not only spread the viral disease but also suck plant saps and damage plant growth. Heavy insecticide spray is being used to control this pest at different plant growth stages. Many such insecticide sprays are not safe for humans and lead to pollution as they persist for a long time in the environment (Zikankuba et al., 2019). Another approach is to identify/develop resistant germplasm, which could be high-yielding and will be the source of resistance in future breeding programs (Snehi et al., 2015). The identification of these resistant cultivars is of high importance as these cultivars have less impact on the cost of production as compared to insecticide sprays, which are more expensive for the control of vectors transmitting these viruses (Habib et al., 2007). This approach was applied in mungbean to identify different resistant cultivars against YMD (Habib et al., 2007). The marker-assisted breeding approach was also used to screen and identify the YMD-resistant and high-yielding mungbean cultivars, which helped the farmers to grow mungbean (Binyamin et al., 2015). The success of conventional breeding for the control of YMD in mungbean encouraged us to acquire soybean germplasm from national and international sources and to identify virus-resistant germplasm. We screened the germplasm for a period of 5 years (2016-2020) under field conditions in the autumn season (August-November) when the incidence of yellow mosaic disease was at its maximum, and successfully identified YMD-resistant germplasm. This would be highly helpful for the soybean breeding program in the future.

The accessions were reduced with selected lines in successive generations by selecting only the HS, R, and HR soybean germplasm, but at the same time, new entries were also added to obtain the best possible gene pool for disease resistance. All the lines were screened at least for three years. During the experiments, the populations of breeding and mutants of locally established cultivars were also developed (results not shown). These plant materials were also tested for YMD resistance (**Table 3**). Initially, the germplasm imported from the USDA along with the local cultivar (NARC-2021) were screened. It has been observed that single plant selection (SPS) is very important for disease resistance in crops (Foxe, 1992). So, for this reason, the SPS was also performed in the same cultivar having high disease severity, and all single plants resistant to YMD were selected and screened in the next generations.

Although symptoms on plant leaves are the initial indication of viruses, these symptoms are not reliable for the exact identification of viral strains or species in plants until the whole genome sequencing of the viral genome is performed (González-Garza, 2017) as the YMD is caused by four different species of LYMVs, namely, MYMIV, MYMV, HgYMV, and DoYMV (Qazi et al., 2007). So, molecular characterization of these species causing YMD in soybeans was needed before further evaluation. For this reason, the total DNA was extracted from both resistant and susceptible soybean samples and was characterized for MYMIV and MYMV. Both the viral species were identified in susceptible cultivars and there was no identification of other LYMVs (**Table 2**). This shows that both species (MYMIV and MYMV) are responsible for the YMD in soybean at the tested location (Faisalabad, Pakistan), and the data have been reported (Rahman et al., 2023a). The sequences for each of the species MYMIV and MYMV were submitted in NCBI with accession nos. MN885463 and MK098184, respectively.

Regarding YMD severity, the highest severity was found in many soybean cultivars such as NBG-22, NBG-31, NBG-47, NBG-117, NARC-2021, SPS-C1, and SPS-C9 (**Table 3**). In 2018, more germplasm were added which were developed by hybridization with local cultivars and mutation. These were given the code CF in cross and M for mutant seeds. The YMD incidence was higher in 2016 and 2019, and lower in 2018 and 2020 (**Figure 2**). The increase in disease incidence in 2019 was due to new soybean entries, whereas the decrease in disease incidence was due to recurrent selections of advanced disease-resistant soybean material throughout the period (2016-2020).

In the studied soybean lines, initially, no germplasm were HR for YMD and there was one single plant in one germplasm (cv. Jack) that was HR to YMD. However, after 2016, many lines showed complete immunity to YMD but the majority of these HR germplasm lost the resistance in the next generations, which shows that, initially, it was pseudo-resistance. Only one line cultivar named “SG-soybean”, an SPS from cv. Jack in the year 2016, maintained complete resistance in respective generations (**Table 3**). This shows that the resistance in the SG-soybean line is stable. Stable resistance is very important in plant breeding programs (Stuthman et al., 2007), although in most cases the resistance is not stable and is lost due to the emergence of new viral strains/species, which is a routine phenomenon that viruses use for their survival and transmission. Hence, this change in the viral genome leads to the production of highly resistant viral strains/pathogens. This has been shown by the appearance of MYMIV in India, which is a novel species of LYMVs. Before the appearance of MYMIV, MYMV was the dominant species that caused YMD, and the resistance was lost due to the emergence of this novel species of MYMIV. However, it could be possible to identify the new resistance source against these two species of LYMVs. Similarly, the resistance break was also observed in cotton for CLCuD by the emergence of new strains of the cotton leaf curl virus, which caused epidemics in Pakistan (Zubair et al., 2017). In tomatoes, it has been observed that the reassortment of the tomato spotted wilt virus led to new strains and broke previously resistant cultivars (Margaria et al., 2015). In our previous investigation, there were many strains and species of begomoviruses identified, including both MYMIV and MYMV (Rahman et al., 2023a; Rahman et al., 2023b), however, the SG-soybean line was found resistant to high disease pressure and there were no symptoms on the SG-soybean line (2016-2020) in all generations (**Figure 4**), although the disease vector, namely, whiteflies, were present in a soybean field, which further strengthens the claim of stable resistance in the SG-soybean line. Second, the presence of MYMIV and MYMV in the field confirms the resistant cultivar having stable resistance. Another possibility of stable resistance could be the presence of the expression of resistance genes that interact and degrade viral proteins. The most resistant protein has been identified for MYMIV in black gram, which interacts with the rep protein of the virus (Patel et al., 2016). The resistance in plants is also governed by gene silencing. It has been found that the resistant cultivar induces viral RNA degradation earlier than the susceptible cultivars after infection (Yadav et al., 2009). Based on previous literature, it is predicted that the SG-soybean line also has some resistance genes that lead to resistance or the cultivar is resistant to whitefly. Further research is needed to determine the mechanism of resistance in the SG-soybean line.

**Figure 7:**
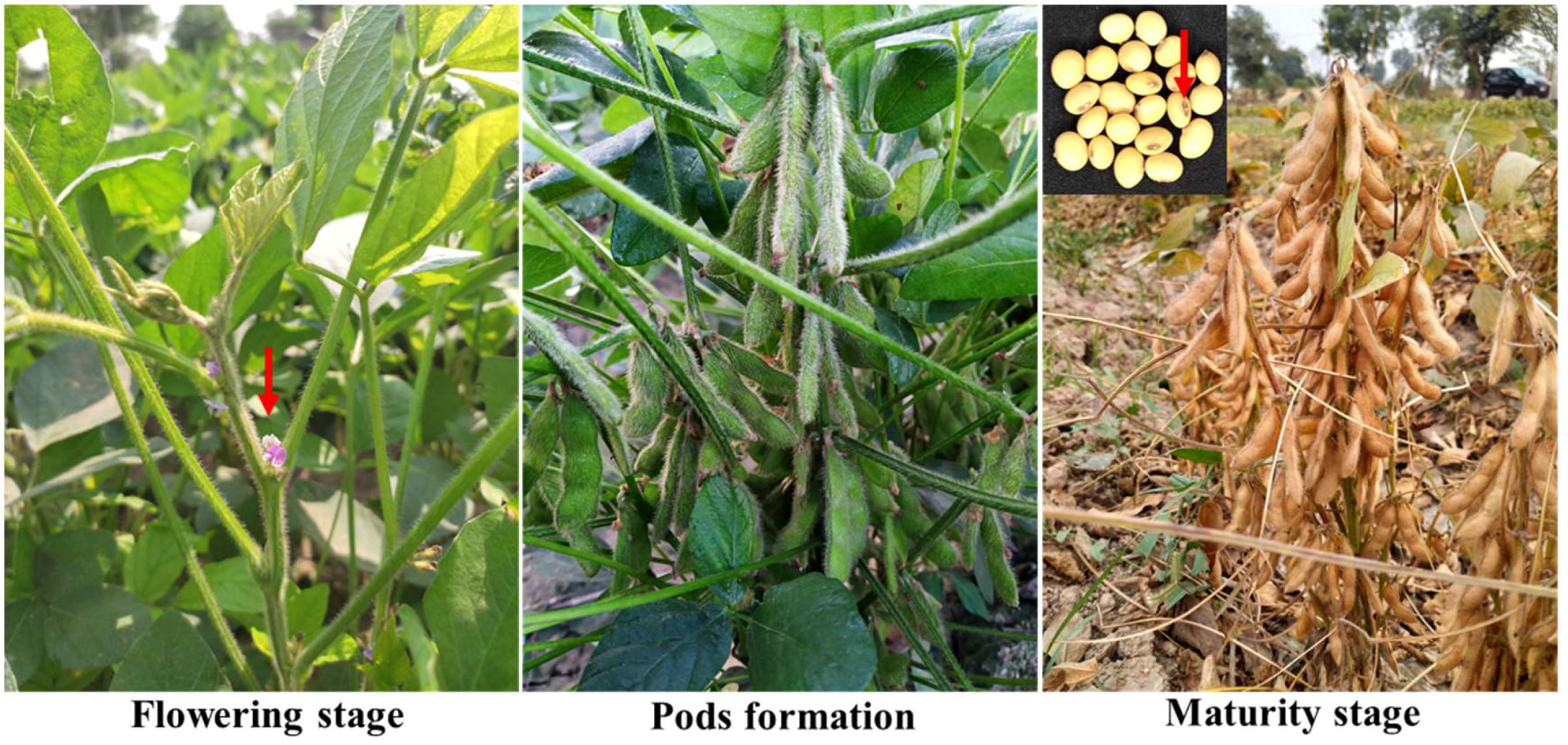
Different growth stages of resistant germplasm, NBG-SG-soybean, in the field.

YMD has a significant impact on plant yield and yield-linked agronomic traits (Baghel et al., 2010). Therefore, in the current study, the impact of YMD on soybeans was recorded based on the agronomic parameters (DF, DM, and PH) and yield-related parameters (PPP, SPP, and GY) of susceptible soybeans compared to resistant soybeans. The potential yield of NARC-2021 is 3000kg/ha. The yield of the SG-soybean line was found to be 7.9 times higher as compared to NARC-2021 and 3 times higher as compared to NBG-117 (**Table 3**). In Pakistan, the Nuclear Institute for Agriculture and Biology (NIAB) produced YMD-resistant cultivars of mungbean. High-yielding mungbean cultivars with susceptibility were crossed with resistant cultivars to further increase the yield (Khattak et al., 2008). This approach could be used to produce YMD-resistant and high-yielding soybean cultivars. The SG-soybean is of short stature (37.3 ± 2.0a cm), having purple flowers, brown pods, golden yellow seeds, off-black hilum, and narrow leaves with dark green color (**Figure 7**). The dark color is a clear indication that the phytochemicals are high, which is of high importance in disease resistance. Further studies are needed to investigate the phytochemical profiles of YMD-resistant and susceptible soybean cultivars.

After the identification of resistant and susceptible cultivars through screening, molecular identification of host genes is very important for a breeding program. There are no such reports in soybeans for YMD resistance genes (Singh et al., 1974). Yadav et al. (2015) identified a SNP that leads to resistance against MYMIV. They found that the mutation, which is a transversion of C to G (*Glyma.18G25100)*, leads to the susceptibility of soybeans to YMD. We hypothesized that the same mutation is responsible for disease susceptibility in tested germplasm. To investigate this, the highly resistant and susceptible genotypes of soybeans were tested for the said mutation. We identified the same SNP in the *Glyma.18G025100* gene on chromosome 18 in the susceptible cultivars; however, this mutation was missing in some susceptible cultivars (**Figure 5B**). It has also been reported that monogenic resistance is of high importance in the initial stages of infection, but in most cases, the monogenic resistance is not durable as the pathogens mutate DNA for their survival (Stuthman et al., 2007).

In 2016, Patel et al. (2016) reported a deletion of 128 amino acids at the start of the dominant *CYR-1* allele that leads to protein truncation and susceptibility to YMD. We, therefore, hypothesized that the ortholog of the *CYR-1* gene is present in soybeans, which may also lead to YMD susceptibility. To test this hypothesis, the sequence of the *CYR-1* gene was aligned with the whole-genome sequence (WGS) of soybeans. We found the closest match to be the 65% sequence similarity of the soybean gene, *Glyma.13G194500*, which was selected for further study. We used overlapping primers to amplify the ortholog in the resistant and susceptible soybean germplasm. Interestingly, we identified a novel deletion of 28 bp in the 5`UTR of the *Glyma.13G194500* gene in only one susceptible soybean accession (**Figure 6C**), while this deletion was not observed in other susceptible cultivars. We named the gene *Glyma.13G194500* as *cyr-1*. This deletion was something unusual, and based on the observation it was expected that the RNA would also be truncated, which would lead to truncated protein or no expression (no protein synthesis). To test this, the total RNA was extracted to synthesize the cDNA, which was used as a template to amplify the *cyr-1* gene transcripts. There was no amplification in the susceptible cultivar, whereas transcripts were amplified in the resistant cultivar. This showed that the RNA is not synthesized in the susceptible cultivar, which may lead to susceptibility in soybeans. Western blotting is also needed to further confirm the absence of the resulting protein.

**Figure 8:**
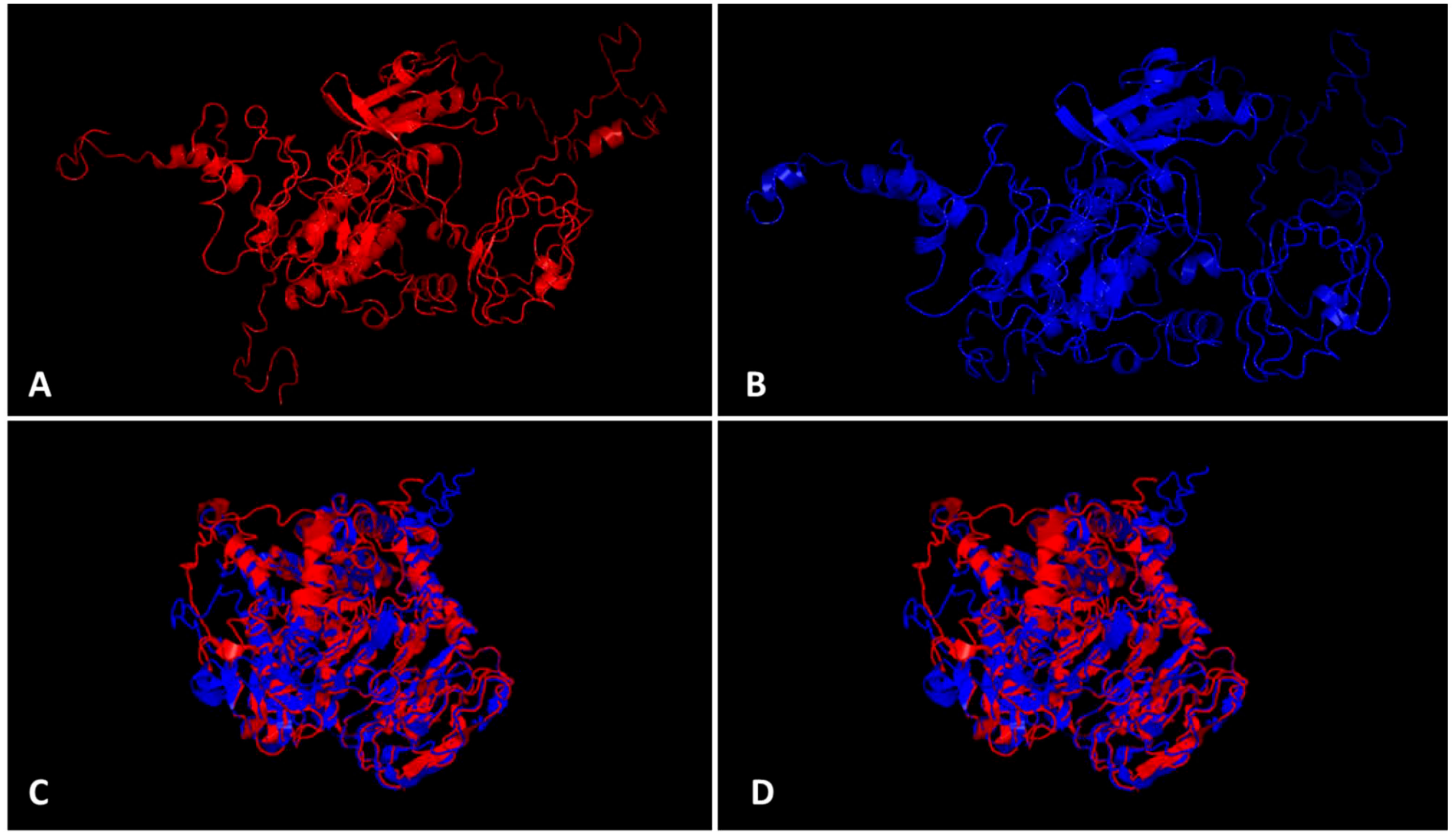
CYR-1 protein structure prediction and alignment. Protein structures identified through I-TASSER visualized in PyMOL **(A)** CYR-1 from resistant and **(B)** CYR-1 from susceptible germplasm, **(C, D)** superimposed and aligned *CYR-1* proteins, which showed a 0.87219 TM-score and root-mean-square deviation (RMSD) score of 3.72, displaying same folds for both proteins.

The bioinformatics analysis by PlantPAN3.0 (Chow et al., 2019) revealed the presence of 14 TFBS spots common in both 5`UTR regions from the CYR-1 gene from resistant and susceptible germplasm, whereas one binding site, bHLH, was additionally found in the 5`UTR region of the CYR-1 gene of resistant germplasm but was absent in the 5`UTR of the CYR-1 gene in susceptible germplasm (**Table S-4**), which reveals the importance of the 5`UTR region in YMD resistance. The ProtParam tool detected slight differences in some parameters of both proteins (Gasteiger et al., 2005). Both proteins have 644 amino acid length, however, resistant CYR-1 has 70805.07 Da molecular weight while susceptible CYR-1 protein has 70779.03 Da. Other features have been highlighted in **Table S-5**. Protein structures obtained from I-TASSER (Zhou et al., 2022) and TM-align (Zhang and Skolnick, 2005) visualized in PyMOL are shown in **Figures 8A–D**. Both proteins from resistant and susceptible germplasm were superimposed and aligned, which showed a 0.87219 TM-score and root-mean-square deviation (RMSD) score of 3.72, displaying the same folds for both proteins. These results conveyed that the proteins with SNP differences only showed minor variations in structure and parameters. Furthermore, the in-silico interaction of CYR-1 both from resistant and susceptible germplasm with the MYMIV viral coat protein exhibited a 0.3537 PSOPIA score (Murakami and Mizuguchi, 2014). Binding affinity in terms of ΔΔG values was detected at the same rate of -10.861 for both proteins through ISLAND (Abbasi et al., 2020), showing no difference in binding affinity of both resistant and susceptible CYR-1 protein to the viral coat protein, which could further be subjected to validation in future studies.

The present investigation has prime importance to uplift soybean research and cultivation in the country. The resistant germplasm could be used to transfer resistance to susceptible cultivars. Identified susceptible and resistant cultivars could also be used as a check in YMD screening experiments. Faisalabad is a hub for YMD incidence under natural field conditions, so the resistant cultivars at this location will be of high importance (Sudha et al., 2013). Moreover, the SNP and *CYR-1* ortholog could be used in marker-assisted breeding to screen YMD-resistant and susceptible soybean germplasm. In the future, the YMD chart (**Figure 1**) developed in the present study can be used for disease scoring in soybeans, facilitating the work of plant virologists to measure disease severity with accuracy.

To the best of our knowledge, this is the first report on the identification of resistant/susceptible cultivars and molecular marker identification against YMD in soybean cultivars.

## Data availability statement

The datasets presented in this study can be found in online repositories. The names of the repository/repositories and accession number(s) can be found in the article/**Supplementary Material**.

## Author contributions

SM, ZM and GR gave the idea, supervised the experiments, and review the final draft of the manuscript. SR, GR, EM and RZN performed the experiments, analyzed the data, and wrote the first draft. The protein *In-silico* study was performed by RZN. IA helped in data analysis. WAP and PL helped in Sanger sequencing of resistant and susceptible soybean germplasm and bioinformatics analysis. MH helped in field data collection. AZG and MSH performed the experiments, analyzed the data, and wrote the first draft of manuscript. Also helped in Sanger sequencing of resistant and susceptible soybean germplasm and bioinformatics analysis.

## Supplementary material

Supplementary Table 1 Field screening of soybean germplasm for YMD resistance in 2016-2020.

Supplementary Table 2 Primers for YMD screening in the soybean field.

Supplementary Table 3 Primers for the amplification of *CYR-1* and *cyr-1* gene ortholog in soybean.

Supplementary Table 4 TFBSs identified in 5`UTR of *CYR-1* gene in Resistant and susceptible germplasm.

Supplementary Table 5 CYR1 proteins’ physio-chemical features.
