# Supplementary material for: A source of resistance against yellow mosaic disease in soybeans correlates with a novel mutation in a resistance gene
Source: Front Plant Sci. 2023 Nov 24;14:1230559. doi: 10.3389/fpls.2023.1230559 (PMC10704482; doi:10.3389/fpls.2023.1230559)
Supplement: Supplementary Table 1 — Field screening of soybean germplasm for YMD resistance in 2016-2020. [file Table_1.docx]

**-Supplementary Tables-**

**Table S-1**

| **Year** | **Genotypes** | |
| --- | --- | --- |
|  | **Highly Resistant** | **Highly Susceptible** |
| **2016** | SG-soybean, SW-31, NBG-8, NBG-109, NBG-57, NBG-59, NBG-62, NBG-66, NBG-69, NBG-70, NBG-230, NBG-197, NBG-23, NBG-103, NBG-281, NBG-284, NBG-174, NBG-84, | NBG-13, NBG-186, NBG-180, NBG-65, FRR-7, NBG-226, NBG-227, NBG-233, NBG-21, NBG-22, NBG-25, NBG-27, NBG-77, NBG-82, NBG-95, NBG-86, NBG-89, NBG-173, NBG-178, NBG-285, NBG-17, NBG-41, NBG-42, NBG-45, NBG-46, NBG-51, NBG-195, SW-19, SW-22, SW-30, NBG-21, NBG-22, NBG-25, NBG-27, NBG-77, NBG-82, NBG-95, NBG-86, NBG-89, NBG-173, NBG-178, NBG-285, NBG-17, NBG-41, NBG-42, NBG-45, NBG-46, NBG-51, NBG-195, SW-19, SW-22, SW-30 |
| **2017** | SG-soybean | NARC-2021*, NBG-47, NBG-103, NBG-106, NBG-105, NBG-108, NBG-117, NBG-153, NBG-186, NBG-187, NBG-193, NBG-195, NBG-302, NBG-197, SW-7, SW-11, SPS-FN1, SW-25, SPS-C1, SPS-C2, SPS-F6, SPS-C8, SPS-F1, SPS-F3, SPS-F4 |
| **2018** | SG-soybean | 24578, 24587, NARC-2021, 24594, 24595, 24598, 24601, 24604, SPS-3, SPS-4, SPS-7, SPS-15, SPS-18, SPS-19, SPS-23, SPS-29, SPS-31, SPS-FN1, C1T0, C1T2, C1T3, C2T1, C2T2, C2T3, C2T5, C3T0, C3T1, C3T2, C3T3, C4T0, C4T1, C4T3, C4T2, C5T0, C5T1, C3T0, C1T3, CIP4F2, C1P3F2, C1P1F2, F XC7, FXCH3C5, FXCH3C3, FXCH3C4, MLU-22,  MLU-38, MLU-40, MLU-42, K24, SW-7, SW-11, SW-28, SUN-1, AV-7, AV-4, SAR-401, PGA-24, PGA-34, PGA-50, PGA-57, PGA-86, PGA-91, PGB-100, PGB-20, PGB-22, PGB-24, PGB-41, CH1, CH2, CH3, CH4, CO-222, NBG-22, NBG-31, NBG-42, NBG-44, NBG-46, NBG-47, NBG-74, NBG-85, NBG-95, NBG-100, 24607, 24611, 24613, 28571, 28572, 28573, 28574, 28578, 28579, 24581, 24582, 32782, SPS-F1, SPS-F3, SPS-F6, SPS-C1, SPS-C2, SPS-C3, SPS-C5, SPS-C7, NBG-106, NBG-117, NBG-130, NBG-195, NBG-207, NBG-216, NBG-274, NBG-302, NBG-346, 3755, 3782, 3792, 17423, 17424, 17432, 17438, 17439, 17440, 17441, 17452, 17458, 23795, 23803, C1T1, C5T3, C5T1, C5T0, C5T6, C2T0, 23924, 23931, 23960, 23981, 23996, 24488, 24490, 24491, 24492, 24498, 24500, 24501, 24504, 24508, 24512, 24513, 24514, 24516, 24517, 24518, 24522, 24527, 24528, 24531, 24532, 24534, 24535, 24536, 24539, 24561, 24562, 24563, 24564, 24566, 24567, 24572, 24573 |
| **2019** | SG-soybean, 24594, GP-1 | NBG-22, NBG-106, NBG-117, NBG-302, NBG-108, NBG-47, NBG-74, NBG-195, NBG-85, NBG-104, NBG-105, NBG-107, NBG-109, NBG-110, NBG-111, NBG-212, NBG-210, NBG-209, NBG-205, NBG-202, NBG-201, NBG-303, NBG-304, NBG-305, NBG-307, NBG-309, NBG-310, NBG-311, NBG-55, 24554, 24557, 24562, 24567, 24573, , 24598, 24611, 32782, SPS-F3, SPS-C1, SPS-F5, SPS-F4, SPS-A1, SPS-F1, SPS-F2, SPS-C9, SPS-C7, CH-1, CH-3, Y, KTG, KPS, KSP, MLU-30, M-44, , MLU-34, MLU-42, MLU-49, , GP-15, GP-16, GP-26, GP-29, GP-30, GP-31, GP-33, GP-36, GP-38, GP-39, GP-40, GP-41, GP-43, GP-44, , GP-50, GP-53, GP-62, GP-65, GP-107, GP-136, GP-150, GP-172, GP-181, GP-203, GP-207, GP-209, GP-215, GP-218, GP-221, GP-222, AV-4, SW-25, SW-28, KY-1, SAR-105, NAI-02, NAI-03, NAI-12, NAI-22, NAI-33, NAI-43, NAI-45, NAI-46, NAI-48, M-5, M-8, M-9, M-13, M-35, M-36, M-53, M-54, M-68, M-69, M-70, M-71, M-75, M-77, M-79, M-80, M-95, M-98, M-118, M-122, M-124, M-125, M-134, M-137, M-170, M-175, M-181, M-183, M-207, M-209, M-210, PGRB-20, PGRB-24, PGA-24, PGV-25, PGA-27, PGB-41, PGB-44, PGA-50, PGB-54, PGB-68, PGA-86, PGA-91, PGB-98, C1P4F2(P34), C1P4F2(P78), C1P4F2(P38), C1P4F2(P83), C1P1F2(P13), C1P1F2(P15), C1P1F2(P16), C1P1A1(P1), C1P1A1(P2) |
| **2020** | SG-soybean | NARC-2021, CH3, SPS-C1, AV-4, PGB-41, J-PK, PGV-25, NBG-YM, MLU-30, NBG-22, SPS-C9, SPS-F1, SAC-105, SUN, NBG-M-186, GP-222, GP-26-28, C1P1A1(P10), C1P1A1(P8), C1P3F2(P9), C1P4F2(P31), C1P4F2(P27), NBG-M-183, NBG-M-79, NBG-M-71, NBG-M-189, MLU-44 |

**Table S-2**

| Primer Code | Primer Pair | Virus Strain |
| --- | --- | --- |
| MYMIV-F | 5` GTAAAGCTTACATCCTCCACCAAGTGG 3` | MYMIV |
| MYMIV-R | 5` TGTAAGCTTTACGCATAATGCTCAATAC 3` | MYMIV |
| MYMV-F | 5` CTCGAGAATTCCGCATTCTTTGAAGCC 3` | MYMV |
| MYMV-R | 5` CTCGAGCCTCAAAGAACCACT 3` | MYMV |

**Table S-3**

| Primer code | Sequence |
| --- | --- |
| 13-G194500-F1  13-G194500-R1 | 5` CTGGAGTCAACGCTTGAAGT 3`  5` GCCGCTTGCCTTGAAAACA 3` |
| 13-G194500-F2  13-G194500-R2 | 5` TGCTTCTACCGTGCGATCAA 3`  5` ATGCCAACAAGTGGGAGAGT 3` |
| 13-G194500-F3  13-G194500-R3 | 5` GGCGCTTTATCATGCATGACC 3`  5` TGGTTCGGATTCCAGTTCCA 3` |
| 13-G194500-F4  13-G194500-R4 | 5` AGCTATCGATTGGGGAGCTG 3`  5` CGGACAAGAATCAATGCGCA 3` |
| 13-G194500-F5  13-G194500-R5 | 5` ATGGTCACCACATGGAAGCA 3`  5` AGACCCTCCTCTGGTAAGCA 3` |
| 13-G194500-F6  13-G194500-R6 | 5` GGGTTGTTGCCACTCTCTCT 3`  5` AACCGTGCAAAGGGTAAGAC 3` |
| 13-G194500-F7  13-G194500-R7 | 5` TTGCCATTGGGAGTTATGGG 3`  5` TCACTCCTCCACTTTGTGCT 3` |
| 13-G194500-F8  13-G194500-R8 | 5` ATGGATACCTTCCTAGCTGCAG 3`  5` TCTGGTCCAGTCTTGTGCTT 3` |

**Table S-4**

| **PlantPAN Matrix ID** | **Transcription factor binding sites (TFBSs) Family** | **Position in 5`UTR of Resistant germplasm** | **Position in 5`UTR of susceptible germplasm** | **Strand** | **Similar Score** | **Hit Sequence** |
| --- | --- | --- | --- | --- | --- | --- |
| TFmatrixID_0131 | AT-Hook | 72 | 44 | - | 1 | TTTATtcgc |
| TFmatrixID_0134 | AT-Hook | 69 | 41 | - | 0.98 | TTTTTtattc |
| **TFmatrixID_0174** | **bHLH** | **40** | **Absent** | **+** | **0.75** | **GCACTtcc** |
| TFmatrixID_0283 | Homeodomain | 17 | 17 | - | 1 | tcAATCAttc |
| TFmatrixID_0284 | Homeodomain | 17 | 17 | - | 1 | tcAATCAttc |
| TFmatrixID_0286 | Homeodomain | 17 | 17 | - | 1 | tcAATCAt |
| TFmatrixID_0289 | Homeodomain | 17 | 17 | - | 1 | tcAATCAttc |
| TFmatrixID_0291 | Homeodomain | 17 | 17 | - | 0.98 | tcAATCAt |
| TFmatrixID_0295 | Homeodomain | 15 | 15 | - | 1 | agtcAATCAt |
| TFmatrixID_0298 | Homeodomain | 18 | 18 | - | 1 | cAATCAttca |
| TFmatrixID_0299 | Homeodomain | 17 | 17 | - | 0.99 | tcAATCAt |
| TFmatrixID_0382 | NAC; NAM | 14 | 14 | + | 1 | gaGTCAAtc |
| TFmatrixID_0445 | WRKY | 14 | 14 | + | 1 | gaGTCAAt |
| TF_motif_seq_0064 | (Motif sequence only) | 60 | 32 | + | 0.73 | CCTTTcttctt |
| TF_motif_seq_0449 | (Motif sequence only) | 12 | 12 | - | 0.75 | ttgAGTCA |

**Table S-5**

| **Parameters** | ***CYR1* protein Resistant germplasm** | ***CYR1* protein Susceptible germplasm** |
| --- | --- | --- |
| **Number of amino acids** | 644 | 644 |
| **Molecular weight** | 70805.07 | 70779.03 |
| **Theoretical pI** | 5.93 | 5.93 |
| **Aliphatic index** | 94.60 | 94.75 |
| **Grand average of hydropathicity (GRAVY)** | -0.116 | -0.111 |
| **Carbon (C)** | 3159 | 3157 |
| **Hydrogen (H)** | 4988 | 4986 |
| **Nitrogen (N)** | 854 | 854 |
| **Oxygen (O)** | 940 | 940 |
| **Sulfur (S)** | 26 | 26 |
